# *CERK1* is required for chitin-triggered reactive oxygen species generation in melon and is broadly conserved in cucurbits

**DOI:** 10.1080/15592324.2025.2578279

**Published:** 2025-11-18

**Authors:** Chun Yu Suen, Hiroki Matsuo, Chujia Jin, Ru Zhang, Akira Mine, Yoshitaka Takano

**Affiliations:** a Laboratory of Plant Pathology, Graduate School of Agriculture, Kyoto University, Kyoto, Japan

**Keywords:** Chitin, CERK1, cucurbits, immunity, ROS

## Abstract

CHITIN ELICITOR RECEPTOR KINASE 1 (*CERK1*), originally identified in *Arabidopsis thaliana*, encodes a pattern recognition receptor that perceives the fungal cell wall component called chitin to activate immune responses, including the production of reactive oxygen species (ROS) against fungal pathogens. Functional *CERK1* orthologs have been identified in plants, such as tomato, rice, and wheat. However, the knowledge of chitin-triggered immunity in Cucurbitaceae plants is currently limited. This study revealed that chitin triggers ROS generation in melon (*Cucumis melo*) and cucumber (*Cucumis sativus*), indicating that chitin is recognized by cucurbits. A subsequent homology search using the Arabidopsis CERK1 sequence identified CERK1 ortholog candidates of melon (CmCERK1) and cucumber (CsCERK1). Virus-induced gene silencing of *CmCERK1* severely reduced chitin-triggered ROS generation in melon, indicating that *CmCERK1* is essential for chitin recognition and the subsequent immune response. Genomic PCR of *CmCERK1* and ROS assay upon chitin treatment in multiple melon commercial cultivars also showed that functional *CmCERK1* is conserved in all the tested cultivars. Further analysis of the available genomes of various cucurbit plants suggested that *CERK1* is broadly conserved in cucurbit plants.

## Introduction

Like animals, plants also possess a sophisticated immune system to combat various kinds of pathogens. Plants can detect pathogens by the recognition of signal molecules either generated or secreted from pathogens.^1^^,^^2^ The plant's immune system consists of a two-tiered innate system that deploys plasma membrane-localized receptors and intracellular immune receptors.^3-5^ A pattern recognition receptor (PRR) is a plasma membrane-localized receptor protein that perceives pathogen-associated molecular patterns (PAMPs), also known as microbe-associated molecular patterns, and initiates PAMP-triggered immunity (PTI).^6^ In response to this defense, pathogens may overcome PTI by secreting a bunch of virulence-related proteins called effectors to suppress the host's immune response.^7^ Plants then evolved a counteracting measure, i.e., intracellular nucleotide-binding/leucine-rich repeat receptors that directly or indirectly recognize pathogen effectors and induce another resistance response called effector-triggered immunity.^8^ Plant PRRs are either receptor-like kinase (RLK) or receptor-like protein (RLP).^9^^,^^10^ For example, LRR-RLK is comprised of a leucine-rich repeat ligand-binding ectodomain, a transmembrane domain, and an intracellular kinase domain, while LRR-RLP shares the same overall structure but lacks the intracellular kinase domain.^11^

Chitin, a major component of fungal cell walls and the exoskeleton of insects, provides structural stability and is crucial to their survivability.^12^^,^^13^ However, chitin is recognized by plants via PRR as a PAMP to trigger PTI.^14^ The first identified chitin-binding PRR was the LysM-RLP, which is called CEBiP in rice.^15^ Subsequently, chitin elicitor receptor kinase 1 (CERK1) was identified in *Arabidopsis thaliana*, *Nicotiana benthamiana*, rice, and wheat.^16-19^ OsCERK1 does not directly bind chitin, while OsCEBiP is responsible for chitin recognition^20^. Interestingly, AtCEBiP can bind chitin but is not required for chitin recognition in Arabidopsis.^21^ AtLYK5 has also been proposed to be the major chitin-binding protein in Arabidopsis with its high affinity to chitin oligomers, while AtCERK1 can bind chitin with modest affinity. ^22-24^ Both AtCERK1 and OsCERK1 are indispensable for initiating the intracellular signaling pathway upon chitin recognition.^14^^,^^16^^,^^18^^,^^22^^,^^25^ The recognition of chitin results in the activation of immune responses, including the rapid accumulation of reactive oxygen species (ROS).^15^^,^^16^ Intriguingly, the chitin-binding receptor was also identified in mice, suggesting the conservation of the chitin recognition mechanism among different eukaryotes.^26^

The Cucurbitaceae family comprises 96 genera and 1000 species, including some well-known commercial crops: cucumber (*Cucumis sativus*), melon (*Cucumis melo*), watermelon (*Citrullus lanatus*), pumpkin and squash (*Cucurbita* spp).^27^ Cucurbits, which have been cultivated worldwide for centuries, are recognized as nutritious and tasty crops with potential in the promotion of personal health.^28-30^ However, cucurbits are prone to several kinds of disease, which results in substantial losses in yield and quality,^31^ and there is currently very limited knowledge about the immune system in cucurbits. Moreover, Guo et al.^32^ suggested that the domestication of cucurbits may have resulted in the loss of disease resistance genes. Consistent with this idea, we recently reported that many commercial melon cultivars lack the *FLS2* gene (named *CmFLS2*), which encodes a PRR essential for the recognition of bacterial flagellin as a PAMP, and suggested that the deleted *FLS2* locus expanded after domestication.^33-35^

In this study, we first investigated the generation of ROS triggered by chitin in both cucumber and melon to investigate whether cucurbits recognize chitin as a PAMP, similar to reported plants. We found that both cucumber and melon rapidly induced ROS generation upon chitin treatment, indicating their recognition of chitin and subsequent induction of the immune response. We then tried to identify the *CERK1* homologs in both cucumber and melon by using the amino acid sequence of *A. thaliana* CERK1. As a result, we identified candidates for potential *CERK1* orthologs in both cucurbitaceous plants. The candidate in melon is identical to *CmCERK1*, which was previously suggested to be involved in chitin-triggered immunity against the cucurbit powdery mildew *Podosphaera xanthii* (syn. *Sphaerotheca fuliginea*), based on studies on several secreted proteins of this pathogen. ^36-39^ However, there is still no direct evidence that *CmCERK1* is essential for chitin-triggered ROS generation in melon. Therefore, we performed a virus-induced gene silencing (VIGS) assay of *CmCERK1* in melon. Importantly, we found that chitin-triggered ROS generation is largely attenuated in *CmCERK1*-silenced melon plants compared with the nonsilenced melon plants, indicating the essential role of *CmCERK1* in chitin recognition and subsequent induction of the immune response. We then experimentally investigated the conservation of *CmCERK1* in commercial melon cultivars and discovered that *CmCERK1* is conserved, in contrast to the case of *CmFLS2.* Further investigation of *CERK1* homologs in cucurbits also revealed that *CERK1* is broadly conserved in cucurbits.

## Results

### Chitin-triggered ROS accumulation and identification of *CERK1* homologs in both melon and cucumber

**Figure 1.**
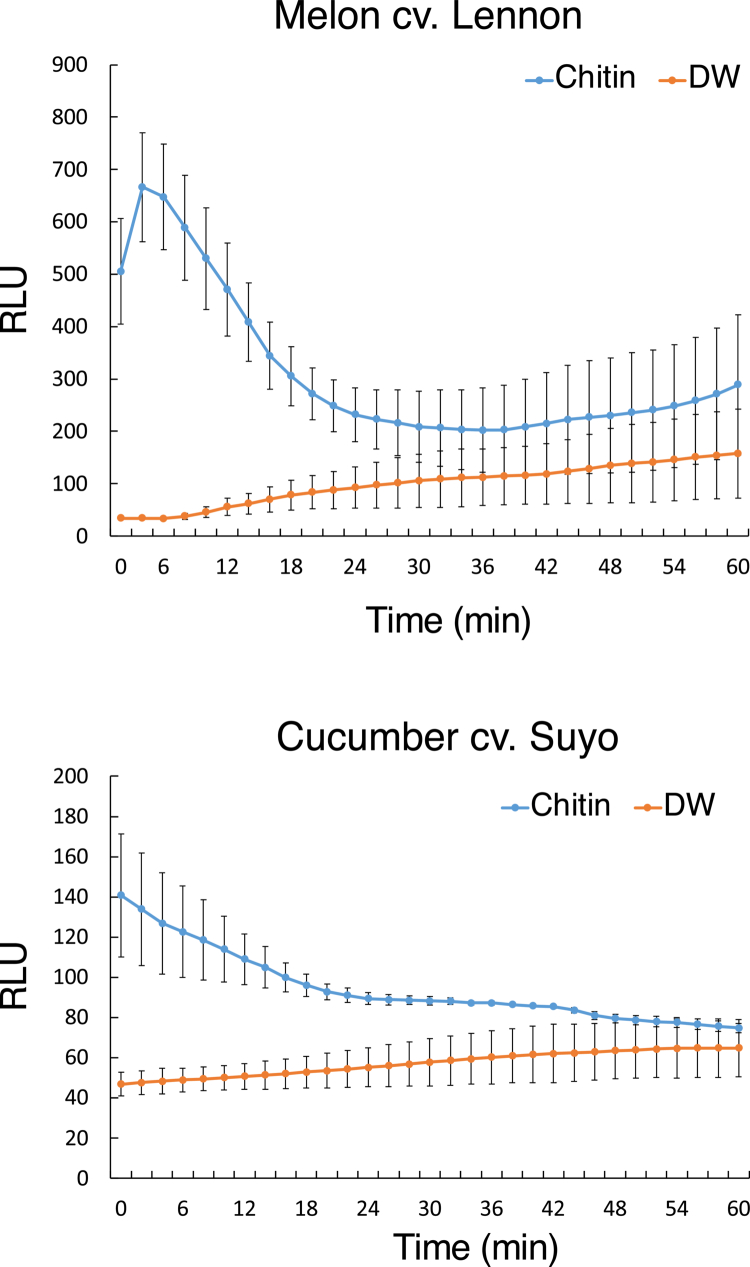
Chitin-triggered ROS production in melon and cucumber. The ROS burst was measured for 60 min in the melon cultivar Lennon (upper) and cucumber cultivar Suyo (lower). Leaf discs after treatment with 400 μg/mL chitin (chitin) or distilled water (DW). Melon data were collected from three plants for each treatment (*n* = 3), and cucumber data were collected from two plants for each treatment (*n* = 2). The values are the means ± SDs. Total ROS production is represented as relative luminescence units (RLUs). Similar results were obtained from two additional experiments.

**Figure 2.**
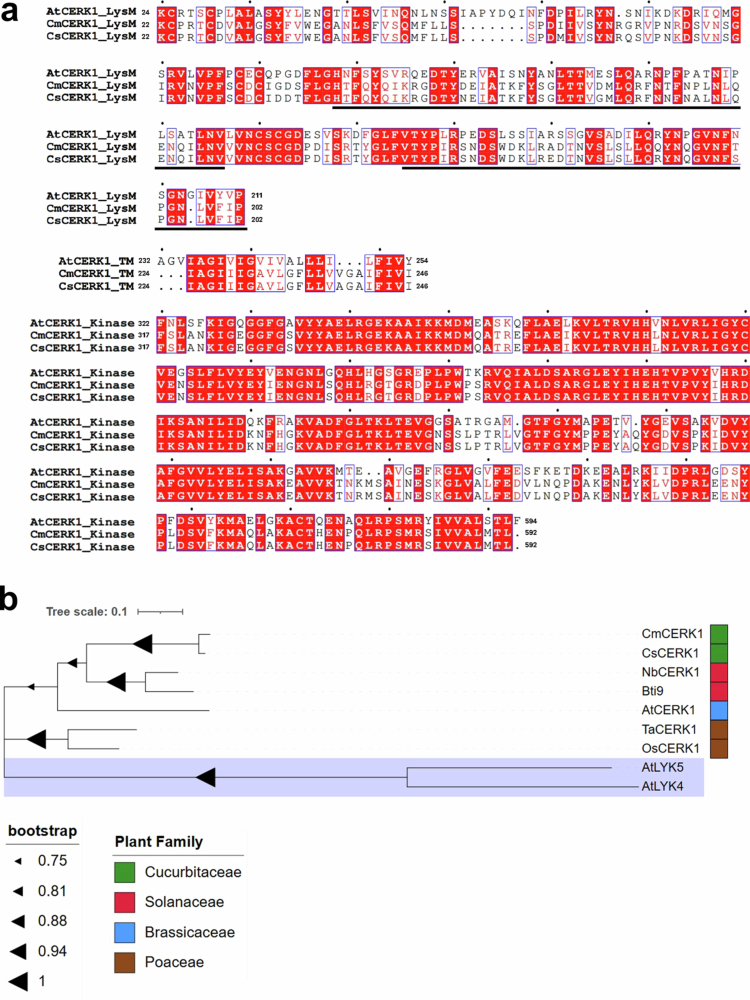
Characterization of CmCERK1 and CsCERK1. (a) Sequence alignment of the domains of AtCERK1, CmCERK1, and CsCERK1. The numbers represent the position of each amino acid in the protein. The sequence underlined with a black line indicates the predicted LysM motif identified by InterProScan. (b) Phylogenetic tree of CERK1 orthologs in seven plant species. The tree was constructed via the maximum likelihood method using OsCERK1 (*Oryza sativa*), TaCERK1 (*Triticum aestivum*), AtCERK1 (*Arabidopsis thaliana*), Bti9 (*Solanum lycopersicum*), NbCERK1 (*Nicotiana benthamiana*), CsCERK1 (*Cucumis sativus*), and CmCERK1 (*Cucumis melo*) genes. AtLYK4 and AtLYK5 were used as outgroups. Triangle-shaped symbols on branches are percent support values out of 100 bootstrap replicates. Only bootstrap values greater than 75% support are shown.

After the identification of *CERK1* in *A. thaliana*, *CERK1* homologs have been characterized in other plants, such as rice, wheat, tomato, and *N. benthamiana.*^17-19^^,^^40^ To investigate whether cucurbits possess functional homologs of *CERK1*, we first investigated whether chitin could trigger immune responses in two cucurbit cultivars, melon (cv. Lennon) and cucumber (cv. Suyo). To monitor the chitin-triggered immune response, we investigated the generation of ROS upon chitin treatment. As a result, we detected a clear increase in ROS accumulation in the chitin-treated plants compared with the distilled water-treated plants (Figure 1), strongly suggesting that both cucurbit plants can recognize chitin and subsequently activate the ROS accumulation. Based on this finding, we next searched for homologs of *CERK1* in both melon and cucumber via BLASTP searches against the NCBI database. The search identified three candidate *CERK1* homologs in cucumber (XP_011658694.1, XP_031744928.1, and XP_031744927.1) and one candidate in melon (MELO3C018836.jh1). The identified melon candidate is identical to the *CmCERK1* reported previously, although there is no direct evidence that *CmCERK1* is essential for chitin-triggered ROS accumulation.^37^^,^^39^ Subsequent manual annotation of three cucumber homologs suggested that they are probably isoforms*.* Because XP_011658694.1 shows the highest sequence similarity to CmCERK1, we assumed that the gene XP_011658694.1 is the CERK1 homolog in cucumber, named CsCERK1. The putative amino acid sequence of the identified CsCERK1 has a percentage identity of 56.77% with the amino acid sequence of AtCERK1, whereas CmCERK1 shares 56.45% identity with AtCERK1. Domain analysis of CmCERK1 and CsCERK1 using InterProScan revealed two LysM domains, one transmembrane domain, and one Ser/Thr kinase domain (Figure 2a). Furthermore, the highest similarity was observed in the kinase domain (72.79%) among the three domains (Figure 2a). Based on these findings, we predicted that both CmCERK1 and CsCERK1 are functional CERK1 orthologs in cucurbit plants. Phylogenetic analysis using CsCERK1 and CmCERK1 with functionally characterized CERK1, i.e., AtCERK1, NbCERK1, Bti9 (tomato CERK1), OsCERK1 (rice CERK1), and TaCERK1 (wheat CERK1), suggested that CsCERK1 and CmCERK1 have the strongest relationships with each other and are mostly close to Bti9 and NbCERK1 and grouped with AtCERK1, whereas OsCERK1 and TaCERK1 are grouped together (Figure 2b). This result is consistent with an earlier report suggesting that *CERK1* orthologs exhibit clear genetic differentiation in monocot and dicot plants.^41^

### Silencing of *CmCERK1* reduced the chitin-triggered ROS accumulation in melon

**Figure 3.**
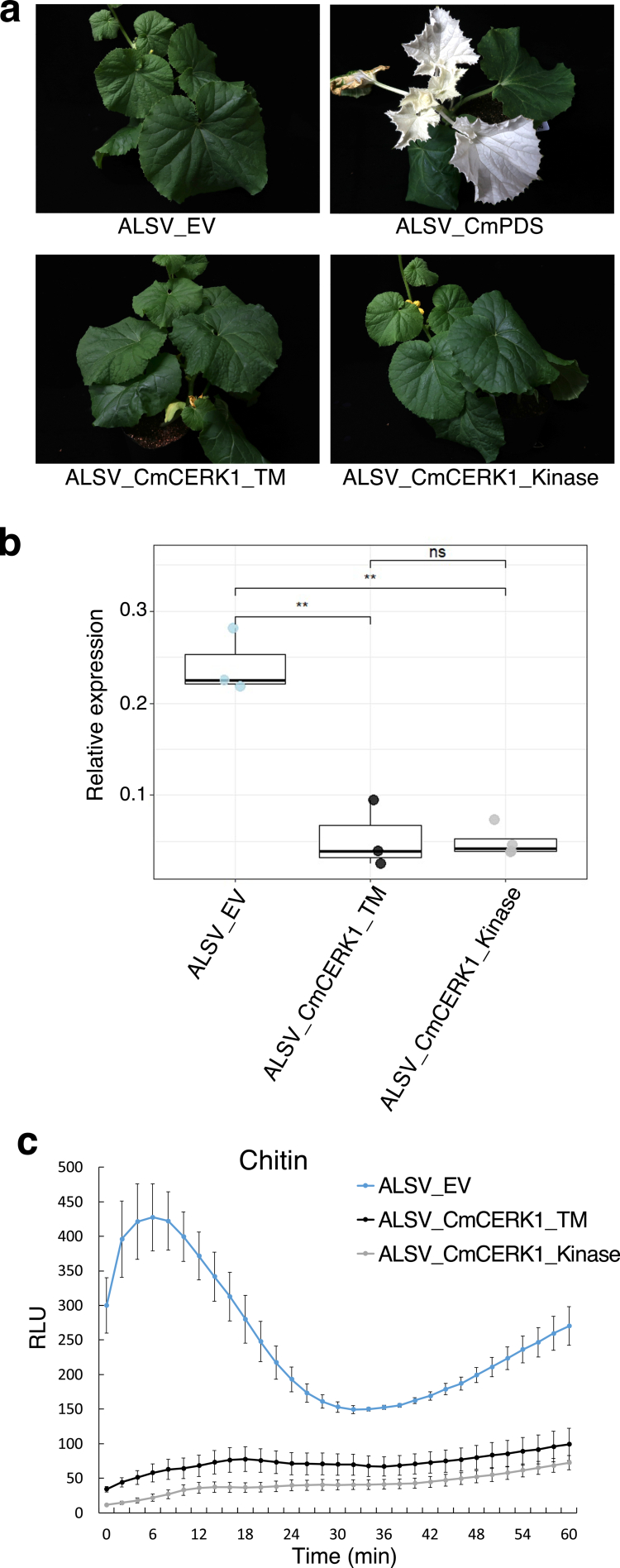
Functional validation of *CmCERK1*. (a) Plant growth comparison between melon cv. Lennon infected with ALSV-EV, ALSV_CmPDS, ALSV_CmCERK1_TM, and ALSV-CmCERK1_Kinase. The photo was taken 28 d after agroinfiltration. Similar results were obtained from three additional experiments. (b) RT‒qPCR analysis of *CmCERK1.* The expression of *CmCERK1* transcripts was measured by RT-qPCR using the fifth true leaf (counting from bottom to top, excluding cotyledons) of ALSV-EV-, ALSV-CmCERK1_TM-, and ALSV-CmCERK1_Kinase-infected melon cv. Lennon and primers specific for *CmCERK1*. Melon *actin* was used as the internal control in the reaction for normalization of the gene expression level. The data were collected from three plants for each treatment (*n* = 3). The data represent the mean ± SD. **p < 0.01. Similar results were obtained from two additional experiments. (c) ROS burst detection for 60  min using melon cv. Lennon leaf disks collected from the fifth true leaf of ALSV-EV-, ALSV-CmCERK1_TM-, and ALSV-CmCERK1_Kinase-infected plants following treatment with 400  μg/mL chitin. The data were collected from three plants for each treatment (*n* = 3). The values are the means ± SDs. ROS production is represented as RLU. Similar results were obtained from three additional experiments.

To assess whether *CmCERK1* is responsible for the generation of chitin-triggered ROS detected in melon (cv. Lennon), VIGS of *CmCERK1* was performed in Lennon using *apple latent spherical virus* (ALSV) vector.^35^^,^^42^^,^^43^ The efficiency of VIGS in Lennon was examined via infiltration of Agrobacterium carrying ALSV_CmPDS as reported previously.^35^ Complete bleaching of leaves based on the silencing of *CmPDS* was observed starting from the 3rd to the 4th leaves; thus, we considered complete silencing of the target gene (*CmCERK1*) starting at the 3rd to the 4th leaves (Figure 3a). Based on the previous report on a study of *TaCERK1* (wheat *CERK1*),^19^ two DNA sequences of *CmCERK1* derived from the TM domain region and the kinase domain region were used for the construction of two silencing vectors, ALSV_CmCERK1_TM and ALSV_CmCERK1_Kinase, respectively. Compared with plants infiltrated with the control empty vector ALSV_EV, plants infiltrated with Agrobacterium harboring ALSV_CmCERK1_Kinase or ALSV_CmCERK1_TM presented no observable difference in growth (Figure 3a). Leaf disks were collected from the 5th leaves of the agroinfiltrated melon plants and subjected to reverse transcription quantitative polymerase chain reaction (RT‒qPCR) of *CmCERK1* and a reactive oxygen species (ROS) assay under chitin treatment. First, RT‒qPCR analysis of *CmCERK1* revealed that the transcript level of *CmCERK1* was significantly reduced in the tested *CmCERK1-*silenced samples compared to the samples from ALSV_EV-infiltrated plants, suggesting that the use of ALSV_CmCERK1_TM and ALSV_CmCERK1_Kinase can effectively knock down the expression of *CmCERK1* in melon (cv. Lennon) (Figure 3b). Importantly, the ROS assay revealed that the accumulation of ROS in all *CmCERK1*-silenced samples was largely abolished upon chitin treatment, whereas the samples infiltrated with ALSV_EV still presented a high level of ROS generation (Figure 3c). These results indicated that the identified *CmCERK1* is the functional ortholog of *CERK1* and is responsible for the chitin-triggered immune response in the melon cultivar.

### *CERK1* is highly conserved in cucurbitaceous plants

**Figure 4.**
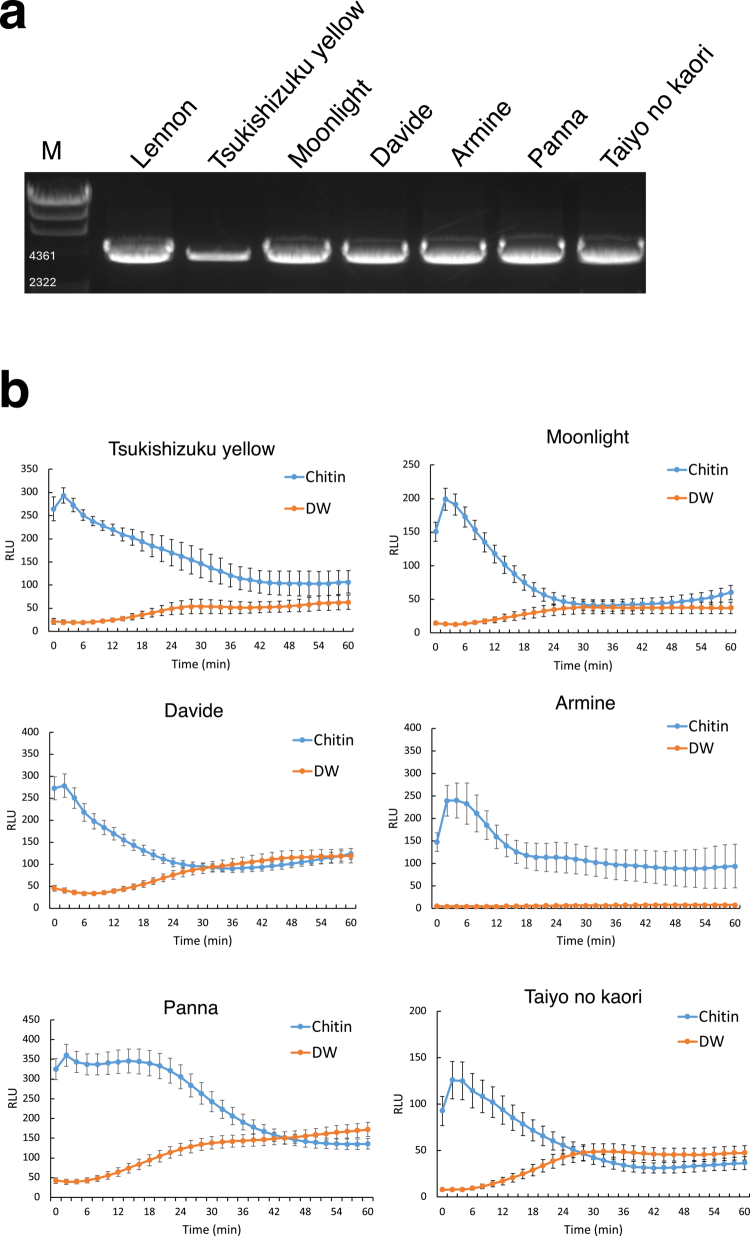
Genotyping of the *CmCERK1* locus and chitin-triggered ROS detection in commercial melon cultivars. (a) Genomic PCR of *CmCERK1* in commercial melon cultivars. Melon (cv. Lennon) was used as a control. Genomic PCR analysis revealed that all the tested commercial melon cultivars conserved a full-length *CmCERK1* band. (b) Chitin-triggered ROS burst assay in melon cultivars. The ROS burst was measured for 60 min using leaf discs of the different cultivars after treatment with 400 μg/mL chitin. The data were collected from 12 individual leaf disks from a single plant of each cultivar. The values are the means ± SDs. ROS production is represented as RLU. Similar results were obtained from one additional experiment.

**Figure 5.**
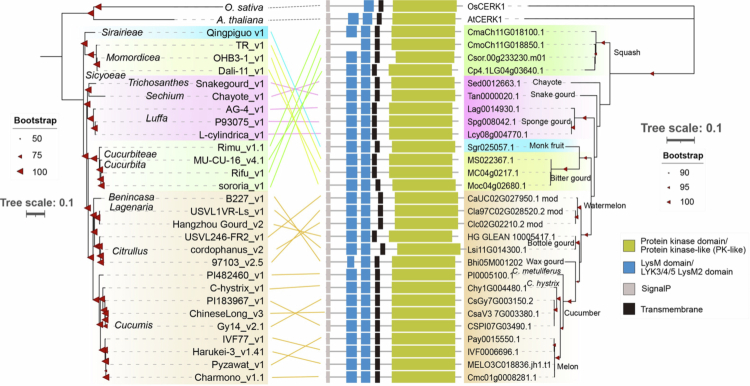
Conservation of CERK1 among Cucurbitaceae. The left panel shows a maximum likelihood phylogenetic tree of assemblies in the Cucurbitaceae family (highlighted by color-coded clades), as well as *O. sativa* and *A. thaliana*. Bootstrap values are indicated by triangles on the nodes, with a larger symbol representing higher support (≥50, ≥75, =100). The right panel presents a phylogenetic tree of the CERK1 homologs identified in Cucurbitaceae. Bootstrap values are indicated by a triangle on the nodes, with a larger symbol representing higher support (≥90, ≥95, =100). The predicted domains of each CERK1 homolog are shown next to their corresponding gene ID. Each homolog contains a protein kinase (or protein kinase-like) domain, LysM domains, a signal peptide (SignalP), and a transmembrane region. The colored lines connect the corresponding assemblies between their domain architecture and the phylogenetic trees.

As mentioned, we previously reported that some commercial melon cultivars lack *CmFLS2*, which encodes a PRR crucial for recognition of the bacterial PAMP flg22.^35^ This finding suggests that immune-related genes are lost upon domestication, resulting in reduced immunity against pathogens. Therefore, we investigated whether *CmCERK1* is conserved in other melon commercial cultivars. Based on the *CmCERK1* genomic sequence, we designed a pair of primers to amplify the full-length *CmCERK1* of different commercial melon cultivars via genomic PCR. The genomic DNA of Lennon was used as a control. We selected seven cultivars with different sugar contents, flesh colors and fruit surface characteristics to check for the conservation of *CmCERK1*. We found that genomic PCR using the designed primers commonly amplified an approximately 4.2 kb band in all seven tested commercial cultivars, including Lennon (Figure 4a). Direct sequencing of the PCR product also confirmed that the amplified product corresponded to *CmCERK1.* Next, we performed a ROS assay in the six tested melon cultivars upon chitin treatment. As a result, all cultivars showed the clear induction of ROS production under the chitin treatment but not under the water treatment (Figure 4b), which is common in the case of Lennon (Figure 1). These results suggest that the *CmCERK1* and *CmCERK1*-dependent ROS generation pathways are highly conserved among commercial melon cultivars, which contrasts with the case of *CmFLS2*. We next investigated whether *CERK1* homologs are also conserved in other cucurbitaceous plants. We tried to identify CERK1 homologs from 28 released Cucurbitaceae genome assemblies using BLASTP (see more details in the legend of Figure 5). Overall, we identified potential CERK1 homologs with a structure similar to that of CmCERK1 in most of the samples (Figure 5, Table S1), suggesting that the *CERK1* gene was highly and widely conserved during the evolution of cucurbitaceous plants. Phylogenetic analysis of the amino acid sequences of these identified cucurbit CERK1 homologs revealed relationships consistent with those inferred from BUSCO gene-based assemblies, suggesting that CERK1 homologs have diverged in accordance with genus and species (Figure 5). Therefore, we concluded that the *CERK1* gene, which is critical for chitin-triggered ROS generation, is highly conserved in cucurbitaceous plants.

## Discussion

Chitin is not synthesized by plants themselves; therefore, plants have evolved receptors that can recognize chitin as PAMPs of fungal pathogens to trigger immune responses against them. As mentioned, mice can also recognize chitin and trigger inflammatory and immune responses,^44^ suggesting that chitin-triggered immunity is conserved in both kinds of organisms, although both plants and animals possess distinct immune systems.

In this study, we revealed that treatment with chitin induces ROS generation in both cucumber (cv. Suyo) and melon (cv. Lennon) plants, indicating that both cucurbitaceous plants can recognize chitin and then trigger ROS generation. We then identified homologs of Arabidopsis *CERK1* in both cucumber (*CsCERK1*) and melon (*CmCERK1*). Although InterProScan analysis revealed two LysM domains in CmCERK1 and CsCERK1, AtCERK1 has three LysM domains. We consider that InterProScan analysis failed to detect the first LysM domain of both proteins, which is an intrinsic limitation of InterProScan analysis that has been found for other LysM domain proteins.^45^ Thus, it is plausible that CmCERK1 and CsCERK1 have three LysM domains, which was also supported by the finding that the first LysM domain region of AtCERK1 has homology to the corresponding region of CmCERK1 and CsCERK1 (Figure 2a).

Subsequent knockdown analysis of *CmCERK1* via VIGS revealed that *CmCERK1* is essential for chitin-triggered ROS generation in melon (cv. Lennon). As mentioned, we recently revealed that many commercial melon cultivars lack the *FLS2* gene (*CmFLS2*), which is essential for the recognition of bacterial flagellin.^35^ Therefore, we investigated whether other commercial melon cultivars have *CmCERK1.* The results demonstrated that all tested commercial melon cultivars retain *CmCERK1*, in contrast to the case of *CmFLS2.* We further investigated the presence of putative *CERK1* orthologues in other cucurbitaceous plants and found that *CERK1* is strongly conserved in a wide range of cucurbitaceous plants. The strong conservation of *CERK1* in cucurbits may be a result of continuous pressure from fungal diseases such as powdery mildew, downy mildew, and anthracnose.^31^^,^^46-49^

**Figure 6.**
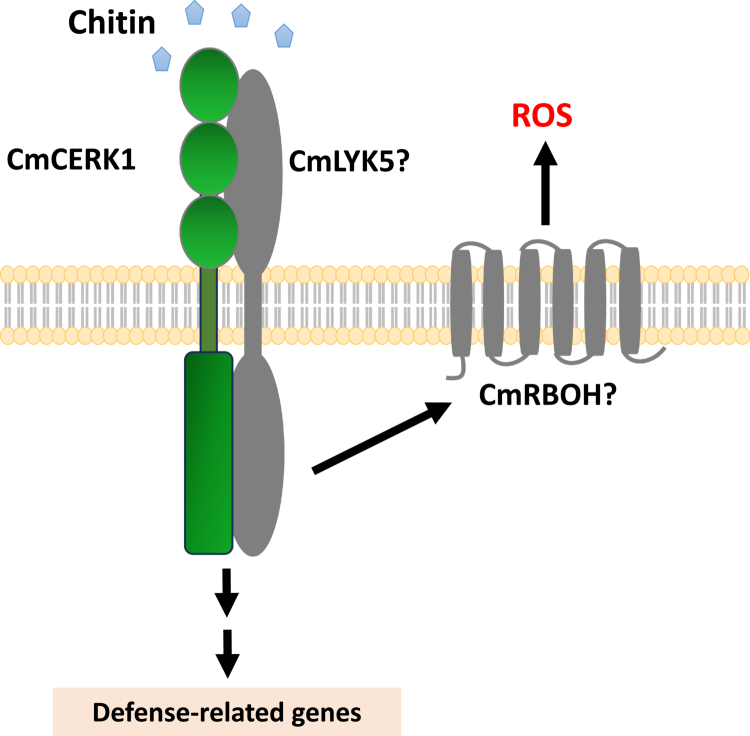
A hypothetical model of the CERK1-mediated defense signaling pathway in melon. CmCERK1 is hypothesized to form a receptor complex with a putative functional homologue of the *Arabidopsis* chitin receptor LYK5, designated CmLYK5, to mediate chitin perception. After chitin is sensed, the proposed CmCERK1-CmLYK5 receptor complex activates an RBOH homolog, termed CmRBOH, leading to the generation of reactive oxygen species (ROS). The receptor complex also activates the expression of defense-related genes. The *CmLYK5* and *CmRBOH* genes have yet to be identified. Future works will aim to identify and characterize these components and decipher the detailed signaling mechanisms underlying CmCERK1-mediated immunity.

As reported previously, AtLYK5, not AtCERK1, is proposed to be the major chitin receptor in *A. thaliana* since AtLYK5 has a relatively high affinity for chitin molecules.^24^ Additionally, rice relies on CEBiP for chitin recognition.^15^ Considering that melons and cucumbers show a relatively close phylogenetic relationship with *A. thaliana* compared to rice, the ortholog of AtLYK5 might exist in both melons and cucumbers. A BLASTP homology search using the AtLYK5 sequence revealed two homologous proteins in melon. One (XP_008440166.1) has 40.87% identity to AtLYK5, and the other (XP_008444671.2) has 35.68% identity. However, the XP_008440166.1 gene also presented the highest degree of homology (46.02%) to AtLYK4. In addition, AtLYK4 is also reported to be an important protein in chitin recognition.^50^ Further studies are necessary to investigate whether XP_008440166.1 is the putative *AtLYK5* ortholog or *AtLYK4* ortholog and to understand its role in chitin recognition in melon (Figure 6).

AtCERK1 and OsCERK1 function as RLKs in the recognition of not only chitin and chitosan but also lipopolysaccharide, peptidoglycan, and β-glucans,^25^^,^^51-55^ suggesting that CERK1 is involved in immunity toward not only fungal pathogens but also bacterial pathogens. This finding also supports our finding that *CERK1* is highly conserved in cucurbits. Interestingly, it was reported that *CERK1* of rice is involved in the recognition of symbiotic signals of arbuscular mycorrhizal fungi.^56^^,^^57^ CERK1 homologs in *Lotus japonicus* (LjLYS6), *Medicago truncatula* (MtCERK1/MtLYK9), and *Pisum sativum* (PsLYK9) have also been reported to be involved in both symbiosis and immunity.^58-60^ It was reported that the inoculation of arbuscular mycorrhizal fungi has beneficial effects on watermelon, cucumber, and squash plants^61-64^; therefore, the possible involvement of cucurbit CERK1 in symbiotic interactions may explain the strong conservation of *CERK1* in cucurbits. In future studies, it is essential to investigate whether *CERK1* of cucurbits, such as *CmCERK1*, is involved in the perception of other microbial signals in addition to chitin.

## Materials and methods

### Plant materials and growth conditions

Cucumber and melon seeds were sown in a mixed soil medium composed of vermiculite, peat moss, and Kon-pals at a 1:1:1 ratio and grown in a growth chamber maintained at 25 °C under a 16-h light/8-h dark photoperiod.

### Measurement of ROS

A luminol-based assay was used to monitor ROS production, following the methods of Keppler (1989). Leaf disks (5 mm in diameter) were punched from the true leaves of each cucurbit species using a cork borer. The disks were incubated overnight in distilled water in the dark. The next day, they were transferred to a 96-well plate containing 50 μL of sterilized distilled water. To assess chitin-induced ROS production, each leaf disk was treated with 50 μL of an assay solution containing 400 μM L-012 (FUJIFILM Wako Chemicals, 120-04891), 20 μg/mL horseradish peroxidase (Sigma-Aldrich, P6782), and 400 μg/mL chitin (Sigma-Aldrich, C9752). Luminescence was measured as relative light units (RLUs) over 60 min using a Luminoskan Ascent (v. 2.1; Thermo Fisher Scientific).

### Plasmid construction

DNA fragments (300 bp) corresponding to the transmembrane (TM) domain and kinase domain of *CmCERK1* were amplified from melon (cv. Lennon) cDNA using gene-specific primer pairs (Table S2). The vector, pBICAL2, and the PCR-amplified insert (CmCERK1_TM and CmCERK1_Kinase) were digested with *Xho*I and *Bam*HI restriction enzymes. Ligation of the amplified fragments into the digested vector was performed using a DNA Ligation Kit (Takara Bio). Three plasmid constructs were generated: ALSV_EV (empty vector), ALSV_CmCERK1_TM, and ALSV_CmCERK1_Kinase.

### ALSV-mediated VIGS

The ALSV-mediated VIGS assay was performed as described previously.^35^ *Agrobacterium* strains containing pBICAL1 were mixed with *Agrobacterium* strains (GV3010) containing ALSV_EV, ALSV_CmCERK1_TM, or ALSV_CmCERK1_Kinase at a 1:1 ratio in infiltration buffer to a final OD_600_ of approximately 0.3. *Agrobacterium* preparations were infiltrated into 8-d-old melon cotyledons using a needleless syringe.

### Quantitative RT‒PCR

Total RNA was isolated from the seventh true leaf (counted from bottom to top) of each sample using the RNeasy Plant Mini Kit (Qiagen). Reverse transcription was performed using the PrimeScript RT Master Mix (Takara Bio). qPCR was conducted with TB Green Premix Ex Taq (Tli RNaseH Plus; Takara) on a CFX Connect Real-Time PCR Detection System (Bio-Rad). The thermal cycling conditions were as follows: initial denaturation at 95 °C for 30 s, followed by 40 cycles of 95 °C for 5 s and 60 °C for 30 s. Lennon-specific primer pairs (Table S2) were used to detect *CmCERK1* expression. Relative gene expression was calculated using the 2^(−ΔCt) method, with *Actin* (MU51303) serving as the internal reference gene for normalization.

### Genotyping of *CmCERK1* in commercial melon cultivars

Genomic DNA was extracted from the cotyledons of each melon plant using the DNeasy Plant Mini Kit (Qiagen), and the bulk DNA of four individuals of each accession was used as the template for genome PCR. Genomic PCR was conducted using the KOD One PCR Master Mix (Toyobo). The thermal cycling conditions were as follows: initial denaturation at 94 °C for 2 min, followed by 35 cycles of 98 °C for 10 s, 55 °C for 5 s, and 68 °C for 25 s. The Sequence_CERK1_F/Sequence_CERK1_R primer pair (Table S2) was used to amplify the *CmCERK1* locus in melon. The PCR products were analyzed via electrophoresis on a 1% agarose gel.

### Phylogenetic analysis of CsCERK1 and CmCERK1 and the conservation of CERK1 homologs among Cucurbitaceae plants

BLASTP was used to identify CERK1 homologs in cucumber and melon using AtCERK1 as the query. All sequences used for the phylogenetic analysis were downloaded from the NCBI database, except Bti9, which was obtained from its original publication.^40^ Sequence alignment was performed using MAFFT^65^ and trimmed with trimAl.^66^ A maximum likelihood (ML) phylogenetic tree (Figure 2b) was constructed using MEGA11 and visualized with the interactive tree of life (iTOL) platform.^67^ Cucurbit CERK1 homologs were identified from the CDS sequences released in CuGenDB v. 2^68^ using AtCERK1 and CsCERK1 as queries in BLAST. In the case of Citrullus CERK1, the original ORF was improved using Helixer^69^ because of incorrect domain annotation. Multiple sequence alignment of the CERK1 homologs was performed using MAFFT,^65^ and an ML phylogenetic tree was constructed using IQ-TREE2^70^ with the Q. plant + G4 model and 1,000 bootstrap replicates. BUSCO v. 5^71^ was used in genome mode with the Embryophyta_odb10 lineage dataset to identify single-copy orthologous genes in cucurbit genome assemblies. Each identified BUSCO gene was aligned using MAFFT and trimmed with trimAl^66^ to remove poorly aligned regions. All trimmed alignments were concatenated, and an ML phylogenetic tree was constructed using RAxML (raxmlHPC-PTHREADS-SSE3) with the PROTGAMMAJTT model and 100 bootstrap replicates.^72^ The resulting phylogenetic trees were visualized using the iTOL platform.^67^

## Supplementary Material

Supplementary material Table S1. CERK1 homolog sequences of the cucurbits used in this study.

Supplementary material Table S2. The list of primiers used in this study.

## References

[1] Sanabria N, Goring D, Nürnberger T, Dubery I. Self/nonself perception and recognition mechanisms in plants: a comparison of self-incompatibility and innate immunity. New Phytol. 2008;178:503–514. doi: 10.1111/j.1469-8137.2008.02403.x. 18346103

[2] Gill US, Lee S, Mysore KS. Host versus nonhost resistance: distinct wars with similar arsenals. Phytopathology. 2015;105(5):580–587. doi: 10.1094/PHYTO-11-14-0298-RVW. 25626072

[3] Zipfel C. Plant pattern-recognition receptors. Trends Immunol. 2014;35:345–351. doi: 10.1016/j.it.2014.05.004. 24946686

[4] Dodds PN, Rathjen JP. Plant immunity: towards an integrated view of plant-pathogen interactions. Nat Rev. Genet. 2010;11:539–548. doi: 10.1038/nrg2812. 20585331

[5] Dangl JL, Horvath DM, Staskawicz BJ. Pivoting the plant immune system from dissection to deployment. Science. 2013;341:746–751. doi: 10.1126/science.1236011. 23950531 PMC3869199

[6] Monaghan J, Zipfel C. Plant pattern recognition receptor complexes at the plasma membrane. Curr Opin Plant Biol. 2012;15:349–357. doi: 10.1016/j.pbi.2012.05.006. 22705024

[7] Toruño TY, Stergiopoulos I, Coaker G. Plant-pathogen effectors: cellular probes interfering with plant defenses in spatial and temporal manners. Ann Rev Phytopathol. 2016;54:419–441. doi: 10.1146/annurev-phyto-080615-100204. 27359369 PMC5283857

[8] Cui H, Tsuda K, Parker JE. Effector-triggered immunity: from pathogen perception to robust defense. Annu Rev Plant Biol. 2015;66:487–511. doi: 10.1146/annurev-arplant-050213-040012. 25494461

[9] Couto D, Zipfel C. Regulation of pattern recognition receptor signaling in plants. Nat Rev Immunol. 2016;16:537–552. doi: 10.1038/nri.2016.77. 27477127

[10] Boutrot F, Zipfel C. Function, discovery, and exploitation of plant pattern recognition receptors for broad-spectrum disease resistance. Annu Rev Phytopathol. 2017;55:257–286. doi: 10.1146/annurev-phyto-080614-120106. 28617654

[11] Smakowska-Luzan E, Mott GA, Parys K, Stegmann M, Howton TC, Layeghifard M, Neuhold J, Lehner A, Kong J, Grünwald K, et al. An extracellular network of *Arabidopsis leucine*-rich repeat receptor kinases. Nature. 2018;553(7688):342–346. doi: 10.1038/nature25184. 29320478 PMC6485605

[12] Brown HE, Esher SK, Alspaugh JA. Chitin: a "hidden figure" in the fungal cell wall. Curr Top Microbiol Immunol. 2020;425:83–111. 31807896 10.1007/82_2019_184

[13] Merzendorfer H, Zimoch L. Chitin metabolism in insects: structure, function, and regulation of chitin synthases and chitinases. J Exp Biol. 2003;206:4393–4412. doi: 10.1242/jeb.00709. 14610026

[14] Gong BQ, Wang FZ, Li JF. Hide-and-seek: chitin-triggered plant immunity and fungal counterstrategies. Trends Plant Sci. 2020;25(8):805–816. doi: 10.1016/j.tplants.2020.03.006. 32673581

[15] Kaku H, Nishizawa Y, Ishii-Minami N, Akimoto-Tomiyama C, Dohmae N, Takio K, Minami E, Shibuya N. Plant cells recognize chitin fragments for defense signaling through a plasma membrane receptor. Proc Natl Acad Sci U S A. 2006;103:11086–11091. doi: 10.1073/pnas.0508882103. 16829581 PMC1636686

[16] Miya A, Albert P, Shinya T, Desaki Y, Ichimura K, Shirasu K, Narusaka Y, Kawakami N, Kaku H, Shibuya N. CERK1, a LysM receptor kinase, is essential for chitin elicitor signaling in *Arabidopsis*. Proc Natl Acad Sci U S A. 2007;104:19613–19618. doi: 10.1073/pnas.0705147104. 18042724 PMC2148337

[17] Segonzac C, Feike D, Gimenez-Ibanez S, Hann DR, Zipfel C, Rathjen JP. Hierarchy and roles of pathogen-associated molecular pattern-induced responses in *Nicotiana benthamiana*. Plant Physiol. 2011;156:687–699. doi: 10.1104/pp.110.171249. 21478366 PMC3177268

[18] Shimizu T, Nakano T, Takamizawa D, Desaki Y, Ishii-Minami N, Nishizawa Y, Minami E, Okada K, Yamane H, Kaku H, et al. Two LysM receptor molecules, CEBiP and OsCERK1, cooperatively regulate chitin elicitor signaling in rice. Plant J Cell Mol Biol. 2010;64:204–214. doi: 10.1111/j.1365-313X.2010.04324.x. PMC2996852 21070404

[19] Lee WS, Rudd JJ, Hammond-Kosack KE, Kanyuka K. *Mycosphaerella graminicola* LysM effector-mediated stealth pathogenesis subverts recognition through both CERK1 and CEBiP homologues in wheat. MPMI. 2014;27:236–243. doi: 10.1094/MPMI-07-13-0201-R. 24073880

[20] Hayafune M, Berisio R, Marchetti R, Silipo A, Kayama M, Desaki Y, Arima S, Squeglia F, Ruggiero A, Tokuyasu K, et al. Chitin-induced activation of immune signaling by the rice receptor CEBiP relies on a unique sandwich-type dimerization. Proc Natl Acad Sci U S A. 2014;111(3):E404–E413. doi: 10.1073/pnas.1312099111. 24395781 PMC3903257

[21] Shinya T, Motoyama N, Ikeda A, Wada M, Kamiya K, Hayafune M, Kaku H, Shibuya N. Functional characterization of CEBiP and CERK1 homologs in *Arabidopsis* and rice reveals the presence of different chitin receptor systems in plants. Plant Cell Physiol. 2012;53:1696–1706. doi: 10.1093/pcp/pcs113. 22891159

[22] Wan J, Zhang XC, Neece D, Ramonell KM, Clough S, Kim SY, Stacey MG, Stacey G. A LysM receptor-like kinase plays a critical role in chitin signaling and fungal resistance in *Arabidopsis*. Plant Cell. 2008;20(2):471–481. doi: 10.1105/tpc.107.056754. 18263776 PMC2276435

[23] Petutschnig EK, Jones AM, Serazetdinova L, Lipka U, Lipka V. The lysin motif receptor-like kinase (LysM-RLK) CERK1 is a major chitin-binding protein in *Arabidopsis thaliana* and subject to chitin-induced phosphorylation. J Biol Chem. 2010;285(37):28902–28911. doi: 10.1074/jbc.M110.116657. 20610395 PMC2937917

[24] Cao Y, Liang Y, Tanaka K, Nguyen CT, Jedrzejczak RP, Joachimiak A, Stacey G. The kinase LYK5 is a major chitin receptor in *Arabidopsis* and forms a chitin-induced complex with related kinase CERK1. eLife. 2014;3: e03766. doi: 10.7554/eLife.03766. 25340959 PMC4356144

[25] Kouzai Y, Mochizuki S, Nakajima K, Desaki Y, Hayafune M, Miyazaki H, Yokotani N, Ozawa K, Minami E, Kaku H, et al. Targeted gene disruption of OsCERK1 reveals its indispensable role in chitin perception and involvement in the peptidoglycan response and immunity in rice. MPMI. 2014;27(9):975–982. doi: 10.1094/MPMI-03-14-0068-R. 24964058

[26] Reese TA, Liang HE, Tager AM, Luster AD, Van Rooijen N, Voehringer D, Locksley RM. Chitin induces accumulation in tissue of innate immune cells associated with allergy. Nature. 2007;447:92–96. doi: 10.1038/nature05746. 17450126 PMC2527589

[27] Chomicki G, Schaefer H, Renner SS. Origin and domestication of Cucurbitaceae crops: insights from phylogenies, genomics and archaeology. New Phytol. 2020;226:1240–1255. doi: 10.1111/nph.16015. 31230355

[28] Salehi B, Capanoglu E, Adrar N, Catalkaya G, Shaheen S, Jaffer M, Giri L, Suyal R, Jugran AK, Calina D, et al. *Cucurbits* plants: a key emphasis to its pharmacological potential. Molecules. 2019;24(10):1854. doi: 10.3390/molecules24101854. 31091784 PMC6572650

[29] Rolnik A, Olas B. Vegetables from the Cucurbitaceae family and their products: positive effect on human health. Nutrition. 2020;78:110788. doi: 10.1016/j.nut.2020.110788. 32540673

[30] Batool M, Ranjha MMAN, Roobab U, Manzoor MF, Farooq U, Nadeem HR, Nadeem M, Kanwal R, AbdElgawad H, Al Jaouni SK, et al. Nutritional value, phytochemical potential, and therapeutic benefits of pumpkin (*Cucurbita* sp.). Plants. 2022;11(11):1394. doi: 10.3390/plants11111394. 35684166 PMC9182978

[31] Grumet R, McCreight JD, McGregor C, Weng Y, Mazourek M, Reitsma K, Labate J, Davis A, Fei Z. Genetic resources and vulnerabilities of major cucurbit crops. Genes. 2021;12:1222. doi: 10.3390/genes12081222. 34440396 PMC8392200

[32] Guo S, Zhang J, Sun H, Salse J, Lucas WJ, Zhang H, Zheng Y, Mao L, Ren Y, Wang Z, et al. The draft genome of watermelon (*Citrullus lanatus*) and resequencing of 20 diverse accessions. Nat Genet. 2013;45:51–58. doi: 10.1038/ng.2470. 23179023

[33] Gómez-Gómez L, Boller T. FLS2: an LRR receptor-like kinase involved in the perception of the bacterial elicitor flagellin in *Arabidopsis*. Mol Cell. 2000;5(6):1003–1011. doi: 10.1016/s1097-2765(00)80265-8. 10911994

[34] Zipfel C, Robatzek S, Navarro L, Oakeley EJ, Jones JD, Felix G, Boller T. Bacterial disease resistance in *Arabidopsis* through flagellin perception. Nature. 2004;428(6984):764–767. doi: 10.1038/nature02485. 15085136

[35] Jin C, Matsuo H, Nakayama Y, Shigita G, Inoue Y, Kato K, Takano Y. A deletion in FLS2 and its expansion after domestication caused global dissemination of melon cultivars defective in flagellin recognition. Plant J Cell Mol Biol. 2024;119(4):1671–1684. doi: 10.1111/tpj.16895. 38924650

[36] Polonio Á, Fernández-Ortuño D, de Vicente A, Pérez-García A. A haustorial-expressed lytic polysaccharide monooxygenase from the cucurbit powdery mildew pathogen *Podosphaera xanthii* contributes to the suppression of chitin-triggered immunity. Mol Plant Pathol. 2021;22:580–601. doi: 10.1111/mpp.13045. 33742545 PMC8035642

[37] Martínez-Cruz JM, Polonio Á, Zanni R, Romero D, Gálvez J, Fernández-Ortuño D, Pérez-García A. Chitin deacetylase, a novel target for the design of agricultural fungicides. J Fungi. 2021;7(12):1009. doi: 10.3390/jof7121009. PMC8706340 34946992

[38] Martínez-Cruz JM, Polonio Á, Ruiz-Jiménez L, Vielba-Fernández A, Hierrezuelo J, Romero D, de Vicente A, Fernández-Ortuño D, Pérez-García A. Suppression of chitin-triggered immunity by a new fungal chitin-binding effector resulting from alternative splicing of a chitin deacetylase gene. J Fungi. 2022;8(10):1022. doi: 10.3390/jof8101022. PMC9605236 36294587

[39] Bakhat N, Jiménez-Sánchez A, Ruiz-Jiménez L, Padilla-Roji I, Velasco L, Pérez-García A, Fernández-Ortuño D. Fungal effector genes involved in the suppression of chitin signaling as novel targets for the control of powdery mildew disease via a nontransgenic RNA interference approach. Pest Manag Sci. 2025;81. doi: 10.1002/ps.8660. 39797552

[40] Zeng L, Velásquez AC, Munkvold KR, Zhang J, Martin GB. A tomato LysM receptor-like kinase promotes immunity and its kinase activity is inhibited by AvrPtoB. Plant J Cell Mol Biol. 2012;69(1):92–103. doi: 10.1111/j.1365-313X.2011.04773.x. PMC3240704 21880077

[41] Yang C, Wang E, Liu J. CERK1, more than a co-receptor in plant-microbe interactions. New Phytol. 2022;234:1606–1613. doi: 10.1111/nph.18074. 35297054

[42] Igarashi A, Yamagata K, Sugai T, Takahashi Y, Sugawara E, Tamura A, Yaegashi H, Yamagishi N, Takahashi T, Isogai M, et al. Apple latent spherical virus vectors for reliable and effective virus-induced gene silencing among a broad range of plants including tobacco, tomato, *Arabidopsis thaliana*, cucurbits, and legumes. Virology. 2009;386:407–416. doi: 10.1016/j.virol.2009.01.039. 19243807

[43] Kawai T, Gonoi A, Nitta M, Kaido M, Yamagishi N, Yoshikawa N, Tao R. Virus-induced gene silencing in apricot (*Prunus armeniaca* L.) and Japanese apricot (*P. mume* Siebold & Zucc.) with the Apple latent spherical virus vector system. J Jpn Soc Hortic Sci. 2014;83(1):23–31. doi: 10.2503/jjshs1.CH-091.

[44] Elieh Ali Komi D, Sharma L, Dela Cruz CS. Chitin and its effects on inflammatory and immune responses. Clin Rev Allergy Immunol. 2018;54:213–223. doi: 10.1007/s12016-017-8600-0. 28251581 PMC5680136

[45] Dallachiesa D, Aguilar OM, Lozano MJ. Improved detection and phylogenetic analysis of plant proteins containing LysM domains. Functional Plant Biology. 2024; 51:FP23131. doi: 10.1071/FP23131. 38007819

[46] Pérez-García A, Romero D, Fernández-Ortuño D, López-Ruiz F, De Vicente A, Torés JA. The powdery mildew fungus *Podosphaera fusca* (synonym *Podosphaera xanthii*), a constant threat to cucurbits. Mol Plant Pathol. 2009;10(2):153–160. doi: 10.1111/j.1364-3703.2008.00527.x. 19236565 PMC6640438

[47] Savory EA, Granke LL, Quesada-Ocampo LM, Varbanova M, Hausbeck MK, Day B. The cucurbit downy mildew pathogen *Pseudoperonospora cubensis*. Mol Plant Pathol. 2011;12(3):217–226. doi: 10.1111/j.1364-3703.2010.00670.x. 21355994 PMC6640371

[48] Shivas RG, Tan YP, Edwards J, Dinh Q, Maxwell A, Andjic V, Weir BS, Liberato JR, Anderson C, Beasley DR, et al. Colletotrichum species in Australia. Australas Plant Pathol. 2016;45:447–464. doi: 10.1007/s13313-016-0443-2.

[49] Matsuo H, Ishiga Y, Kubo Y, Yoshioka Y. *Colletotrichum orbiculare* strains distributed in Japan: race identification and evaluation of virulence to cucurbits. Breed Sci. 2022;72(4):306–315. doi: 10.1270/jsbbs.22011. 36699825 PMC9868334

[50] Wan J, Tanaka K, Zhang XC, Son GH, Brechenmacher L, Nguyen TH, Stacey G. LYK4, a lysin motif receptor-like kinase, is important for chitin signaling and plant innate immunity in Arabidopsis. Plant Physiol. 2012;160:396–406. doi: 10.1104/pp.112.201699. 22744984 PMC3440214

[51] Liu B, Li JF, Ao Y, Qu J, Li Z, Su J, Zhang Y, Liu J, Feng D, Qi K, et al. Lysin motif-containing proteins LYP4 and LYP6 play dual roles in peptidoglycan and chitin perception in rice innate immunity. Plant Cell. 2012;24(8):3406–3419. doi: 10.1105/tpc.112.102475. 22872757 PMC3462640

[52] Ao Y, Li Z, Feng D, Xiong F, Liu J, Li JF, Wang M, Wang J, Liu B, Wang HB. OsCERK1 and OsRLCK176 play important roles in peptidoglycan and chitin signaling in rice innate immunity. Plant J Cell Mol Biol. 2014;80(6):1072–1084. doi: 10.1111/tpj.12710. 25335639

[53] Rebaque D, Del Hierro I, López G, Bacete L, Vilaplana F, Dallabernardina P, Pfrengle F, Jordá L, Sánchez-Vallet A, Pérez R, et al. Cell wall-derived mixed-linked β-1,3/1,4-glucans trigger immune responses and disease resistance in plants. Plant J Cell Mol Biol. 2021;106(3):601–615. doi: 10.1111/tpj.15185. PMC8252745 33544927

[54] Yang C, Liu R, Pang J, Ren B, Zhou H, Wang G, Wang E, Liu J. Poaceae-specific cell wall-derived oligosaccharides activate plant immunity via OsCERK1 during *Magnaporthe oryzae* infection in rice. Nat Commun. 2021;12(1):2178. doi: 10.1038/s41467-021-22456-x. 33846336 PMC8042013

[55] Desaki Y, Kouzai Y, Ninomiya Y, Iwase R, Shimizu Y, Seko K, Molinaro A, Minami E, Shibuya N, Kaku H, et al. OsCERK1 plays a crucial role in the lipopolysaccharide-induced immune response of rice. New Phytol. 2018;217(3):1042–1049. doi: 10.1111/nph.14941. 29194635

[56] Miyata K, Kozaki T, Kouzai Y, Ozawa K, Ishii K, Asamizu E, Okabe Y, Umehara Y, Miyamoto A, Kobae Y, et al. The bifunctional plant receptor, OsCERK1, regulates both chitin-triggered immunity and arbuscular mycorrhizal symbiosis in rice. Plant Cell Physiol. 2014;55(11):1864–1872. doi: 10.1093/pcp/pcu129. 25231970

[57] Zhang X, Dong W, Sun J, Feng F, Deng Y, He Z, Oldroyd GE, Wang E. The receptor kinase CERK1 has dual functions in symbiosis and immunity signalling. Plant J Cell Mol Biol. 2015;81(2):258–267. doi: 10.1111/tpj.12723. 25399831

[58] Bozsoki Z, Cheng J, Feng F, Gysel K, Vinther M, Andersen KR, Oldroyd G, Blaise M, Radutoiu S, Stougaard J. Receptor-mediated chitin perception in legume roots is functionally separable from Nod factor perception. Proc Natl Acad Sci U S A. 2017;114(38):E8118–E8127. doi: 10.1073/pnas.1706795114. 28874587 PMC5617283

[59] Leppyanen IV, Shakhnazarova VY, Shtark OY, Vishnevskaya NA, Tikhonovich IA, Dolgikh EA. Receptor-like kinase LYK9 in *Pisum sativum* L. is the CERK1-like receptor that controls both plant immunity and AM symbiosis development. Int J Mol Sci. 2017;19(1):8. doi: 10.3390/ijms19010008. 29267197 PMC5795960

[60] Gibelin-Viala C, Amblard E, Puech-Pages V, Bonhomme M, Garcia M, Bascaules-Bedin A, Fliegmann J, Wen J, Mysore KS, le Signor C, et al. The Medicago truncatula LysM receptor-like kinase LYK9 plays a dual role in immunity and the arbuscular mycorrhizal symbiosis. New Phytol. 2019;223(3):1516–1529. doi: 10.1111/nph.15891. 31058335

[61] Chen S, Zhao H, Zou C, Li Y, Chen Y, Wang Z, Jiang Y, Liu A, Zhao P, Wang M, et al. Combined inoculation with multiple arbuscular mycorrhizal fungi improves growth, nutrient uptake and photosynthesis in cucumber seedlings. Front Microbiol. 2017;8:2516. doi: 10.3389/fmicb.2017.02516. 29312217 PMC5742139

[62] Wang C, Li X, Zhou J, Wang G, Dong Y. Effects of arbuscular mycorrhizal fungi on growth and yield of cucumber plants. Commun Soil Sci Plant Anal. 2008;39(3-4):499–509. doi: 10.1080/00103620701826738.

[63] Ye L, Zhao X, Bao E, Cao K, Zou Z. Effects of arbuscular mycorrhizal fungi on watermelon growth, elemental uptake, antioxidant, and photosystem II activities and stress-response gene expressions under salinity-alkalinity stresses. Front Plant Sci. 2019;10:863. doi: 10.3389/fpls.2019.00863. 31333702 PMC6616249

[64] Al-Hmoud G, Al-Momany A. Effect of four mycorrhizal products on squash plant growth and its effect on physiological plant elements. Adv Crop Sci Tech. 2017;5(260):10–4172.

[65] Katoh K, Standley DM. MAFFT multiple sequence alignment software version 7: improvements in performance and usability. Mol Biol Evol. 2013;30(4):772–780. doi: 10.1093/molbev/mst010. 23329690 PMC3603318

[66] Capella-Gutiérrez S, Silla-Martínez JM, Gabaldón T. TrimAl: a tool for automated alignment trimming in large-scale phylogenetic analyses. Bioinformatics. 2009;25(15):1972–1973. doi: 10.1093/bioinformatics/btp348. 19505945 PMC2712344

[67] Letunic I, Bork P. Interactive tree of life (iTOL) v6: recent updates to the phylogenetic tree display and annotation tool. Nucleic Acids Res. 2024;52(W1):W78–W82. doi: 10.1093/nar/gkae268. 38613393 PMC11223838

[68] Yu J, Wu S, Sun H, Wang X, Tang X, Guo S, Zhang Z, Huang S, Xu Y, Weng Y, et al. CuGenDBv2: an updated database for cucurbit genomics. Nucleic Acids Res. 2023;51(D1):D1457–D1464. doi: 10.1093/nar/gkac921. 36271794 PMC9825510

[69] Stiehler F, Steinborn M, Scholz S, Dey D, Weber APM, Denton AK. Helixer: cross-species gene annotation of large eukaryotic genomes using deep learning. Bioinformatics. 2021;36(22-23):5291–5298. doi: 10.1093/bioinformatics/btaa1044. 33325516 PMC8016489

[70] Minh BQ, Schmidt HA, Chernomor O, Schrempf D, Woodhams MD, von Haeseler A, Lanfear R. Corrigendum to: IQ-TREE 2: new models and efficient methods for phylogenetic inference in the genomic era. Mol Biol Evol. 2020;37(8):2461–2461. doi: 10.1093/molbev/msaa131. 32556291 PMC7403609

[71] Manni M, Berkeley MR, Seppey M, Simão FA, Zdobnov EM. BUSCO update: novel and streamlined workflows along with broader and deeper phylogenetic coverage for scoring of eukaryotic, prokaryotic, and viral genomes. Mol Biol Evol. 2021;38(10):4647–4654. doi: 10.1093/molbev/msab19. 34320186 PMC8476166

[72] Stamatakis A. RAxML version 8: a tool for phylogenetic analysis and post-analysis of large phylogenies. Bioinformatics. 2014;30(9):1312–1313. doi: 10.1093/bioinformatics/btu033. 24451623 PMC3998144

